# Ultrasound Enhanced Delivery of Molecular Imaging and Therapeutic Agents in Alzheimer**'**s Disease Mouse Models

**DOI:** 10.1371/journal.pone.0002175

**Published:** 2008-05-14

**Authors:** Scott B. Raymond, Lisa H. Treat, Jonathan D. Dewey, Nathan J. McDannold, Kullervo Hynynen, Brian J. Bacskai

**Affiliations:** 1 The Harvard-MIT Division of Health Sciences and Technology, Cambridge, Massachusetts, United States of America; 2 Department of Neurology, Massachusetts General Hospital, Harvard Medical School, Boston, Massachusetts, United States of America; 3 Department of Radiology, Brigham and Women's Hospital, Harvard Medical School, Boston, Massachusetts, United States of America; 4 Duke University School of Medicine, Durham, North Carolina, United States of America; 5 Sunnybrook Health Sciences Centre, University of Toronto, Toronto, Canada; Mental Health Research Institute of Victoria, Australia

## Abstract

Alzheimer's disease is a neurodegenerative disorder typified by the accumulation of a small protein, beta-amyloid, which aggregates and is the primary component of amyloid plaques. Many new therapeutic and diagnostic agents for reducing amyloid plaques have limited efficacy in vivo because of poor transport across the blood-brain barrier. Here we demonstrate that low-intensity focused ultrasound with a microbubble contrast agent may be used to transiently disrupt the blood-brain barrier, allowing non-invasive, localized delivery of imaging fluorophores and immunotherapeutics directly to amyloid plaques. We administered intravenous Trypan blue, an amyloid staining red fluorophore, and anti-amyloid antibodies, concurrently with focused ultrasound therapy in plaque-bearing, transgenic mouse models of Alzheimer's disease with amyloid pathology. MRI guidance permitted selective treatment and monitoring of plaque-heavy anatomical regions, such as the hippocampus. Treated brain regions exhibited 16.5±5.4-fold increase in Trypan blue fluorescence and 2.7±1.2-fold increase in anti-amyloid antibodies that localized to amyloid plaques. Ultrasound-enhanced delivery was consistently reproduced in two different transgenic strains (APPswe:PSEN1dE9, PDAPP), across a large age range (9–26 months), with and without MR guidance, and with little or no tissue damage. Ultrasound-mediated, transient blood-brain barrier disruption allows the delivery of both therapeutic and molecular imaging agents in Alzheimer's mouse models, which should aid pre-clinical drug screening and imaging probe development. Furthermore, this technique may be used to deliver a wide variety of small and large molecules to the brain for imaging and therapy in other neurodegenerative diseases.

## Introduction

Neurodegenerative diseases are difficult to treat, in part because of the blood-brain barrier (BBB), a composite of highly specialized endothelial and perivascular structures that limit transport of molecules into the brain. Medicinal chemists must extensively modify drugs in order to bypass the BBB, at significant cost and delayed time to clinic. Many large biological molecules, such as antibodies and siRNA, cannot be easily modified to cross the BBB passively. Although several promising strategies have been developed for harnessing endogenous active transport systems [1], [2], these techniques have limited carrying capacity and must be tailor-made for the application. A generic, non-invasive technique to deliver drugs to the brain would allow preclinical efficacy screening and facilitate basic research with biological agents.

We and others have shown that low-intensity, focused ultrasound (FUS) administered with microbubbles (MB) (here termed FUS-MB therapy), can transiently disrupt the blood-brain barrier [3], [4]. At frequencies suitable for non-invasive trans-skull focusing [5], FUS-MB causes BBB-disruption only in the transducer focal volume, which can be selected in real-time by magnetic resonance (MR) imaging for precise anatomical delivery. FUS-MB has been used to deliver antibodies, including Herceptin [6], [7] and small-molecule chemotherapeutics [8], in wild-type animals. To date, FUS-MB has not been applied to neurodegenerative disorders, a broad class of diseases that is largely refractory to current diagnostic and therapeutic approaches.

Alzheimer's disease (AD) is a progressive and fatal neurodegenerative disorder that afflicts millions worldwide and has no cure. AD pathogenesis is putatively tied to the accumulation of misfolded proteins, beta-amyloid (Aβ) and hyperphosphorylated tau, which aggregate and form amyloid plaques and tau tangles [9]. Emerging diagnostic and therapeutic strategies focus on detecting and reducing Aβ aggregates [10], [11]. One promising strategy for amyloid plaque clearance is immunization against Aβ, either by active vaccination with Aβ or by administration of anti-Aβ antibodies, termed passive immunization [12]. Screening anti-Aβ antibodies in transgenic AD mouse models generally requires high, repeated dosing over months, which is enormously expensive due to the cost of purified antibody and transgenic mouse strains. We believe that FUS-MB might alleviate some of this cost by allowing localized delivery of anti-Aβ antibodies at high concentration.

In undertaking this study, we were concerned that differences between aged transgenics and wild-type mice might affect FUS-MB. For example, aged transgenic mice (often >1 year old) generally have thicker, more brittle skulls and altered vascular physiology. Additionally, we wanted to use a simple benchtop sonication system to allow high throughput FUS-MB for drug studies. Here we demonstrate that FUS-MB enhances both small-molecule and antibody delivery in transgenic AD mice and can be achieved without MRI guidance in a simple benchtop setup.

## Results

### MRI-Guided Focused Ultrasound-Microbubble Treatment

We initially attempted small-molecule delivery using a proven MRI-guided sonication protocol [7]. For a targeted imaging probe, we chose Trypan blue, a bis-azo red fluorescent dye that shares structural similarity with Congo red (Figure S1) and also binds amyloid [13]. Normally, Trypan blue does not cross the BBB because of its size (MW, 916 Da) and hydrophilicity (4 sulfonic acid groups). When the BBB is disrupted, Trypan blue extravasates into tissue, resulting in a grossly visible blue stain [14], and thus has been used classically to demonstrate BBB breakdown.

**Figure 1:**
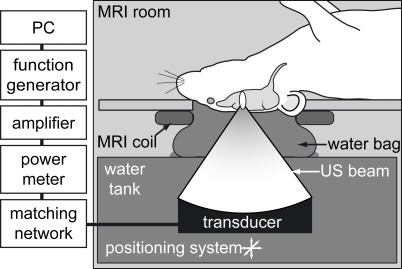
Schematic of focused ultrasound system. Mice were placed supine on the animal platform with the head circled by a transmit/receive surface MRI coil (constructed in-house); ultrasound was coupled to the brain from the tank via a water bag. The transducer was positioned with an xyz positioning system and driven with a PC-triggered, RF-amplified, function generator.

**Figure 2:**
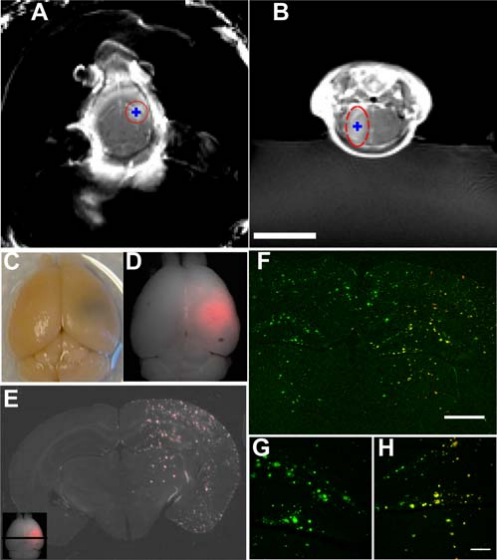
MRI-guided focused ultrasound-microbubble treatment. Transgenic mice were sonicated at a single location determined from pre-treatment MR imaging and received intravenous MR-contrast agent and Trypan blue, an Aβ-targeting red fluorophore, after sonication. (A–B) T1-weighted, contrast-enhanced MR images taken 5 minutes following FUS treatment. Intended sonication locations are indicated by blue “+”. Enhancing volume noted with red. (C–D) Post-mortem brain; the sonication location is visible as a blue spot in right hemisphere on photography (C) and red fluorescence (D) from Trypan blue staining. (E) Trypan blue-labeled amyloid plaques appear as punctate red fluorescence staining throughout the sonication location. Inset: black line indicates approximate location of section from whole brain. In (F–H), multiphoton images of 20 µm sections were stained with FITC-conjugated anti-Aβ antibodies (3d6) to confirm Trypan blue staining. Green is FITC-3d6, red is Trypan blue. (G) 10x magnified view of untreated hippocampus. (H) Corresponding treated hippocampus from the same section. Scale bars: B, 1 cm, F, 1 mm, H, 200 µm.

Aged (11–12 month-old), double transgenic mice (n = 2) expressing APPswe and PSEN1dE9 mutations were treated with MRI-guided FUS-MB (Figure 1), and then received Trypan blue. BBB-disruption was confirmed in vivo with gadolinium-based, contrast-enhanced MRI (Figure 2). The millimeter-scale anatomical structures selected pre-treatment were correctly targeted and exhibited BBB-disruption on T1-weighted fast spin-echo (FSE) MR images. Gadolinium-based contrast-enhancement was observed in a ∼2 mm×5 mm ellipsoid confined to the right hemisphere and traversing the brain from cortex to thalamus, apparent in the coronal plane (Figure 2). We observed some contrast in the right ventricle, immediately adjacent to the intended target position (blue “+”, Figure 2A–B).

Post-mortem brains exhibited a distinct blue spot, confined to a sub-region approximating the dimensions of the transducer focus, from Trypan blue extravasation across the BBB. No blue staining was grossly observed in the contralateral hemisphere. On 50-µm coronal sections, Trypan blue fluorescence was significantly higher in the treated volume (p = 0.02, paired t-test, comparing treated and untreated regions of interest (ROIs), n = 5 animals including 3 from benchtop experiments below) and appeared to concentrate at small plaque-like inclusions in the cortex, hippocampus, and to a lesser extent, in the thalamus (Figure 2E).

We confirmed that the Trypan blue-stained aggregates were amyloid plaques with immunohistochemistry (IHC) using a FITC-conjugated anti-Aβ antibody (3d6) (Figure 2F). In the sonicated volume, Trypan blue (red) co-localized with FITC-3d6 signal (green) and stained dense core plaques. When normalized for background autofluorescence, average Trypan blue fluorescence was up to 21-fold higher (16.5±5.4, including benchtop experiments from below) in the treated vs. untreated cortex. These results indicate that FUS-MB significantly increased Trypan blue delivery within the sonication volume and that Trypan blue successfully diffused from the blood vessels to an extravascular target, amyloid plaques.

### Aged PDAPP Mice

To test the robustness of FUS-MB across transgenic strains, we repeated the treatment described above in 26 month-old PDAPP mice (n = 2), which express a mutant form of APP that results in severe amyloidosis and accompanying pathology, including neuritic dystrophies, astrocytosis, and microgliosis [15]. We observed BBB disruption on contrast-enhanced, T1-weighted FSE imaging following FUS-MB and from Trypan blue staining in the sonicated hemisphere of post-mortem brains (Figure S2). Thus, FUS-MB was effective in two different transgenic AD models, even in extensively aged mice.

### Benchtop Focused Ultrasound-Microbubble Therapy

We next tested the capabilities of a MRI-free, benchtop system by administering Trypan blue or Evans blue, a Trypan blue isoform (Figure S1), to transgenic mice (n = 6, 1 sham-treated) 10 minutes following FUS-MB therapy. The entire procedure took approximately 5 minutes per animal, compared to 30–45 minutes with the MR-guided procedure. All treated mice had distinct blue staining in a focal region of the right hemisphere on post-mortem examination. We did not observe blue staining in the sham-treated animal. Sonication locations, determined from blue staining, were within 2.2 mm of the intended location and accuracy depended upon the system operator (Figure S3). These results confirmed that FUS-MB is possible in transgenic animals using our simple benchtop sonication system, albeit with poorer localization accuracy.

### Focused Ultrasound-Microbubble Enhanced Antibody Delivery

**Figure 3:**
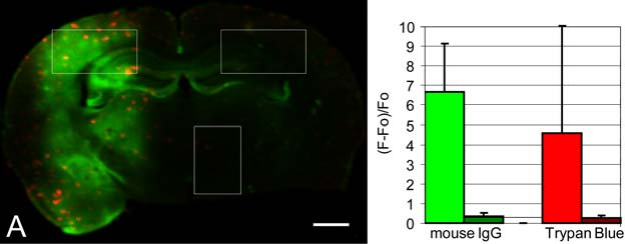
Endogenous mouse IgG extravasation in ultrasound-treated AD transgenic mouse. 50 µm sections from the ultrasound-treated mice described in Figure 2 were stained with Alex 488 conjugated goat anti-mouse IgG antibodies and imaged with a microarray plate scanner. (A) Coronal section through the sonicated volume exhibits Trypan blue (red) and mouse IgG (green) staining. (B) Comparison of average IgG (green) and Trypan blue (red) in treated (bright) and untreated (dark) cortex, normalized to background fluorescence (Fo) in the untreated thalamus. Regions of interest are shown in white in (A). Error bars show the standard deviation for the selected regions of interest. Scale bar: A, 1 mm.

Although small molecule delivery has some application in AD therapy and diagnostics, we were primarily interested in the delivery of much larger biological molecules, such as antibodies, for AD therapy. Two previous results suggested that antibody delivery should be possible in aged transgenic mice. First, experiments by Kinoshita et al demonstrated antibody delivery to young, wild-type mice using FUS-MB [7]. Furthermore, in our first FUS-MB experiments in transgenic AD mice, we saw evidence of endogenous mouse IgG extravasation. When we stained the FUS-MB treated brains with Alexa 488-conjugated goat anti-mouse IgG antibodies (Invitrogen, Eugene, OR, USA), we observed diffuse fluorescence throughout the sonicated volume, 10 to 20-fold greater than in the untreated hemisphere, indicative of endogenous mouse IgG that had extravasated across the BBB (Figure 3A).

**Figure 4:**
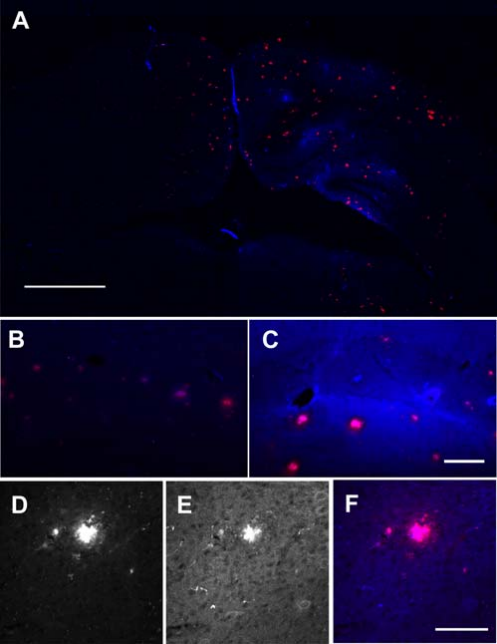
Ultrasound enhanced delivery of anti-Aβ antibodies in a transgenic AD mouse. Alexa Fluor 350 conjugated goat anti-rabbit antibodies (blue fluorescence) were used to detect rabbit anti-Aβ antibodies injected immediately before FUS-MB. (A) The brain shows focal staining in the sonication location that overlaps with Trypan blue-positive plaques (red fluorescence). Alexa Fluor 350 signal is significantly stronger in the treated (C) versus untreated (B) hippocampi. Many plaques show clustered Alexa 350 staining (E) that overlapped with Trypan blue staining (D) (overlay in F). Scale bars: A, 1 mm, C, 100 µm, F, 100 µm.

We next attempted the delivery of Aβ-targeted antibodies by administering rabbit anti-Aβ antibodies (Anti-β-Amyloid (1–40) developed in rabbit, whole antiserum, A 8326, Sigma Aldrich, St. Louis, Missouri) to transgenic AD mice immediately before benchtop FUS-MB. Following perfusion and tissue sectioning, we stained for the rabbit antibody using Alexa Fluor 350 conjugated goat anti-rabbit IgG antibodies (Invitrogen, Eugene, OR, USA). We observed stronger Alexa Fluor 350 fluorescence (blue) in the treated hemisphere (p = 0.06, paired t-test, n = 3, comparing treated and untreated ROIs normalized to control tissue) which coincided with Trypan blue-stained amyloid plaques (see Figure 4).

Alexa Fluor 350 fluorescence did not perfectly match Trypan blue fluorescence and was somewhat heterogeneous throughout the sonicated volume, with stronger fluorescence in the hippocampus and around a few large blood vessels (Figure 4A). In these sub-regions, there was strong background staining, which we interpreted as extravasated, non-specific rabbit IgG, which is the primary constituent of the whole antiserum preparation. On the microscopic level, Alexa Fluor 350 fluorescence colocalized with Trypan blue-positive plaques and vascular amyloid (Figure 4D–F).

### Histological Evaluation

**Figure 5:**
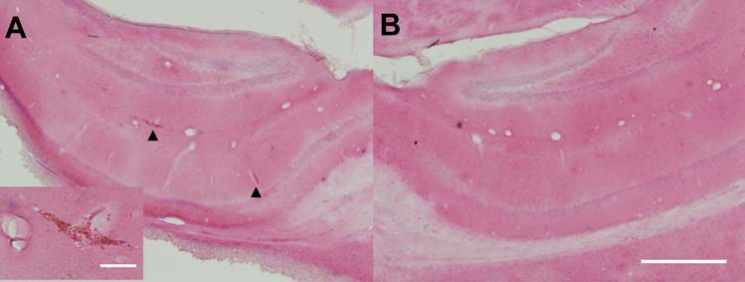
Histological evaluation of FUS-MB treatment. Hemotoxylin and eosin stained tissue sections from the treated volume were examined for signs of tissue damage. We observed scattered petechial hemorrhages in treated regions (A), compared to no damage in untreated regions (B). (A) Treated hippocampus. Arrowheads indicate petechiae. Inset shows a magnified view of the petechiae on left. (B) Untreated hippocampus. Scale bars: A-inset, 50 µm, B, 500 µm.

We used hemotoxylin and eosin (H&E) stained tissue sections to assess FUS-MB induced tissue damage in post-mortem brain sections. 3 of 9 brains had 2–10 scattered petechiae on individual 50-µm H&E sections (Figure 5); 1 of these had petechiae on the inferior aspect of the brain and in the thalamus, along the axis of the transducer. When we compared histological effects with the peak negative pressure applied, we found that the 2 animals treated at high pressure (estimated 0.8 MPa) had petechiae, whereas only 1 of 7 treated at a lower pressure (estimated 0.67 MPa) had petechiae. These results were consistent with other histological studies of FUS-MB damage in rabbit and mice at similar ultrasound parameters [6], [7], 16[].

## Discussion

These experiments demonstrated that ultrasound can enhance the delivery of small fluorescent agents and large biological immunotherapeutics in transgenic mouse models of Alzheimer's disease. Depending on the strain used, transgenic AD mice are aged 9–15 months before they develop amyloid plaques and other neurological features of AD. The young, wild-type mouse skull attenuates the acoustic pressure by 13±7% [6], whereas the transgenic AD mouse skull, considerably thicker and more brittle, could potentially attenuate the ultrasound field even further and might shift the focal coordinate position. Despite these differences between transgenic and wild-type animals, we observed robust Trypan blue delivery in two different transgenic strains for a wide range of ages (9–26 months). This suggests that, at least at the low frequency used here (f = 0.69 MHz), impedance effects from the skull could be overcome.

Additionally, some AD transgenic strains have altered vascular physiology, including increased BBB permeability and risk of hemorrhage and other vascular accidents [17], [18], [19], [20]. Although there was some evidence for low Trypan blue leakage in the untreated hemisphere, FUS-MB increased Trypan blue fluorescence 16.5-fold in the treated region (Figure 3B). Furthermore, we did not see increased histological tissue damage in transgenic mice compared to previous studies with wild-type mice and rabbits. Only 1 of 7 mice in this study treated at the lowest pressure amplitude (0.67 MPa) exhibited petechiae. Thus, FUS-MB delivery of small molecules can be achieved with minimal histological damage and markedly increases brain dosage compared to background BBB “leak” in transgenic AD mouse models.

Similar dosage enhancement was seen for antibodies (Figure 3B). We measured a 10–20 fold fluorescence increase for endogenous IgG in the treated compared to untreated regions and a ∼3-fold fluorescence increase for rabbit anti-Aβ IgG. These estimates were made by comparing average fluorescence from treated and untreated regions of interest on stained brain sections, with normalization to background, untreated tissue, and are therefore only semi-quantitative estimates of relative dose. It is unclear why endogenous IgG leakage was greater than IV-administered antibodies. Because IV-administered antibodies were at a significantly lower concentration than endogenous IgG, it is likely that this discrepancy represents the limited sensitivity of IHC staining techniques, as well as tissue fixation, fluorescence quenching, imaging parameters, and image analysis. However, we cannot rule out inherently different pharmacokinetics of rabbit vs. mouse IgG in a mouse model.

Rabbit anti-Aβ delivery had a heterogeneous distribution within the transducer focus, with enhanced Alexa Fluor 350 staining around large blood vessels and in the hippocampus. This heterogeneity was similar to the delivery distribution observed by Kinoshita et al [7], and might be explained by the limited diffusion of antibodies within the neuropil or the mechanisms of FUS-MB mediated BBB disruption. Electron microscopy studies of FUS-MB enhanced delivery suggest that, following FUS-MB, large molecules cross the BBB via para-endothelial passages (through breached tight junctions) or by trans-endothelial, vesicular transport [21]. These two separate mechanisms were further corroborated by real-time multiphoton imaging that demonstrated kinetically distinct delivery processes [22]. Vesicular transport is preferentially upregulated in arterioles [23], and thus might be heterogeneously activated across brain anatomy depending on the relative density of arterioles. Further work is needed to determine if adequate homogeneity can be achieved for therapeutic efficacy of anti-Aβ antibodies.

Others have suggested that the targeting capabilities of FUS-MB could be applied for drug delivery to small anatomical targets, such as the hippocampus [24]. We successfully targeted amyloid plaques within hippocampus using MRI-guidance, but had some “off-target” delivery in the cortex and thalamus due to the dimensions of our transducer focal volume (∼2 mm×14 mm, ellipsoid). This limitation could be resolved by using a larger diameter transducer with a tighter focal volume.

Targeting with our benchtop system was less accurate. From the cohort described here, targeting was within 2.2 mm of the intended location and varied with the system user (see Figure S3). Variability was likely due largely to animal motion and inaccurate animal positioning. Others have used a more traditional stereo-tactic approach, with the head fixed in ear-bars, and sonication from an applicator [24], [25], with better accuracy.

Because benchtop FUS-MB can be performed with a simple setup in a short procedure (5 min), it offers the possibility for high-throughput anti-Aβ antibody screening. It is unclear from these experiments if a single FUS-MB enhanced anti-Aβ antibody dose is adequate for amyloid clearance. Future work will quantify antibody dose and amyloid clearance in treated animals.

Besides antibodies, FUS-MB may be used to deliver a variety of imaging and therapeutic molecules to the transgenic mouse brain, including newly developed amyloid-targeted MR probes [26], [27] and siRNA [28], [29]. We believe that FUS-MB may be used for imaging and drug delivery in transgenic models of neurodegenerative diseases besides Alzheimer's, and could be used for delivering functional imaging probes to the brain for basic research.

Despite promising advances in immunotherapeutics [17], [30], [31], [32], [33] and PET imaging [34], [35], [36], Alzheimer's disease remains intractable to current therapeutic and diagnostic strategies. By overcoming the BBB, FUS-MB may potentiate agents with demonstrated efficacy and could open new avenues of research by reducing the developmental constraints on new Alzheimer's agents. We view this as a powerful preclinical research tool.

A clinical FUS system composed of a 512-element phased transducer array at a frequency similar to the current study (670 kHz) has been developed for MRI-guided intracranial thermal ablation [5], [37]. We suggest that such a system could feasibly be used at low powers to achieve noninvasive BBB disruption with high spatial resolution for targeted FUS-MB delivery. Before translation to the humans, however, FUS-MB must pass vigorous safety and efficacy benchmarks to prove therapeutic benefit and minimize side-effects. Furthermore, we must better understand the underlying biomechanical mechanisms for FUS-MB therapy. Ongoing work at our institutions is aimed at understanding the cellular response to FUS-MB and demonstrating the therapeutic effects of FUS-MB-enhanced drug delivery for CNS disorders.

## Materials and Methods

### MR-guided FUS-MB Treatment

All animal experiments were performed at Brigham and Women's Hospital under national and institutional guidelines using protocols authorized by the Harvard Medical Area Standing Committee on Animals. Experiments were conducted using an MR-compatible sonication system consisting of an ultrasound transducer submerged in a small tank of degassed, deionized water coupled to an animal platform above (see Figure 1). The transducer was attached to a manual XYZ positioning system to allow fine positioning of the focal volume. Sonication pressure and power were estimated from calibration measurements described previously [6]. The entire sonication system was placed on the MR table and moved into a clinical 3T MR scanner (GE Healthcare, Milwaukee, WI) for imaging. We determined focal volume coordinates by MR thermometry in a gel phantom sonicated at high powers [38].

Transgenic mice (n = 2, 11–12 month old, B6C3-Tg(APPswe, PSEN1dE9)85Dbo/J; n = 2, 26 month old, PDAPP) were anesthetized (70 mg/kg ketamine, 10 mg/kg xylazine) and prepped for treatment by shaving and depilating the head with depilatory lotion. Mice were placed supine on the animal platform and imaged pre-treatment to specify the sonication location coordinates; the transducer focus was then moved using the manual positioner. Mice were sonicated (f = 0.69 MHz, burst length = 10 ms, pulse repetition frequency = 1 Hz, peak negative pressure = 0.67–0.8 MPa, estimated acoustic power = 0.28–0.4 W, exposure length = 40–45 s) with concomitant IV administration of a microbubble contrast agent (0.03–0.05 ml Optison, GE Healthcare, Chalfont, St Giles, UK, or 0.01 ml 1∶10 diluted Definity in saline, Bristol-Myers Squibb, New York, NY) and subsequent BBB-disruption was monitored with T1-weighted fast spin echo MRI following IV administration of gadopentetate dimeglumine (Magnevist, Berlex Laboratories, Inc., Wayne, NJ) administered IV (0.25 ml/4 kg) as a bolus injection. Mice received Trypan blue (0.06 ml, 4%, IV) 10 minutes following FUS-MB. We perfused the mice 3–4 hrs after FUS-MB (5 ml saline and 5 ml 10% phosphate-buffered formalin), and extracted the brains for post-mortem analysis.

### Benchtop FUS-MB Treatment

Similar to above, the transducer was mounted on an XYZ positioning system and submerged in a tank of degassed, deionized water. Ultrasound energy was coupled to the animal above via a mylar window. Before experiments, the transducer was focused onto cross-hairs drawn onto the mylar window, providing a reference for animal alignment. Mice were placed supine with the shaved head directly contacting a mylar window and positioned such that the transducer focus was 4 mm caudal to the eyes and centrally aligned; the transducer was then moved 2–3 mm to the right to place the transducer focus squarely in the right hemisphere.

Transgenic mice (n = 8, 9–12 month old, B6C3-Tg(APPswe, PSEN1dE9)85Dbo/J) were anesthetized and prepped for treatment as described above. Of the 8 mice, 5 were treated and included in study, 1 was sham-treated, and 2 were excluded because they died before or during treatment, putatively from anesthesia. We treated the mice as described above with ultrasound exposure and concurrent IV administration of microbubbles. Some mice (n = 3) received rabbit anti-Aβ antibodies (0.1 ml of 53 mg/ml whole sera, Sigma Aldrich) IV immediately before FUS-MB. Ten minutes following FUS-MB, mice received Trypan blue or Evans blue (0.06 ml, 4%, IV), which are used interchangeably for BBB studies. The mice were perfused 3–6 hrs after FUS-MB as above.

### Tissue Analysis

Extracted brains were photographed (FinePix S7000, Fuji-film, Japan) and imaged using a multi-spectral small animal imaging system (Maestro, CRI, Woburn, MA, USA) to confirm Trypan blue staining. Brains were sectioned at 20 or 50 µm and representative sections were stained using standard IHC or H&E. Prepared sections were mounted and imaged using multiphoton microscopy (Bio-Rad Laboratories,

Hercules, CA, USA) and a microarray scanner (ScanArray Express, Perkin Elmer, Waltham, MA, USA). Images were analyzed using ImageJ [39]. Fluorescence fold changes were calculated by comparing the mean fluorescence in large ROIs from treated and contralateral untreated cortex. Fluorescence counts were normalized to mean signal from untreated thalamus to reduce the artifact from background staining and autofluorescence (so-called Fo in Figure 3).

## Supporting Information

Figure S1 Chemical structures of amyloid imaging fluorophores. (A) Congo red, (B) Trypan blue, and (C) Evans blue.
(0.41 MB TIF)

Figure S2 MRI-guided focused ultrasound-microbubble treatment in PDAPP mice. Aged PDAPP mice were treated with FUS-MB at a location determined from pre-treatment MR imaging and received intravenous MR-contrast agent and Trypan blue, an Aβ-targeting red fluorophore, after sonication. (A–B) T1-weighted, contrast-enhanced MR images taken 5 minutes following FUS treatment. Intended sonication locations are indicated by blue “+”. (C–D) Post-mortem brain; the sonication location is faintly visible as a blue spot in right hemisphere on photography (C) and red fluorescence (D) from Trypan blue staining. Scale bar: B, 1 cm.
(5.40 MB TIF)

Figure S3 Benchtop focused ultrasound-microbubble treatment. Transgenic mice were sonicated at a single location in the right hemisphere using a benchtop sonication system and received Trypan blue or Evans blue IV. 5/5 animals exhibited focal blue staining in the right hemisphere. (a) Focal blue staining from Trypan blue on post-mortem, excised brain. (b) Schematic displaying sonication locations (red and blue dotted lines) from two different system users (red vs. blue) for two intended target locations (red and blue crosses). Scale bar: B, 1 cm.
(5.14 MB TIF)
