# Supplementary figures and images for: Ultrasound Enhanced Delivery of Molecular Imaging and Therapeutic Agents in Alzheimer's Disease Mouse Models
Source: PLoS One. 2008 May 14;3(5):e2175. doi: 10.1371/journal.pone.0002175 (PMC2364662; doi:10.1371/journal.pone.0002175)

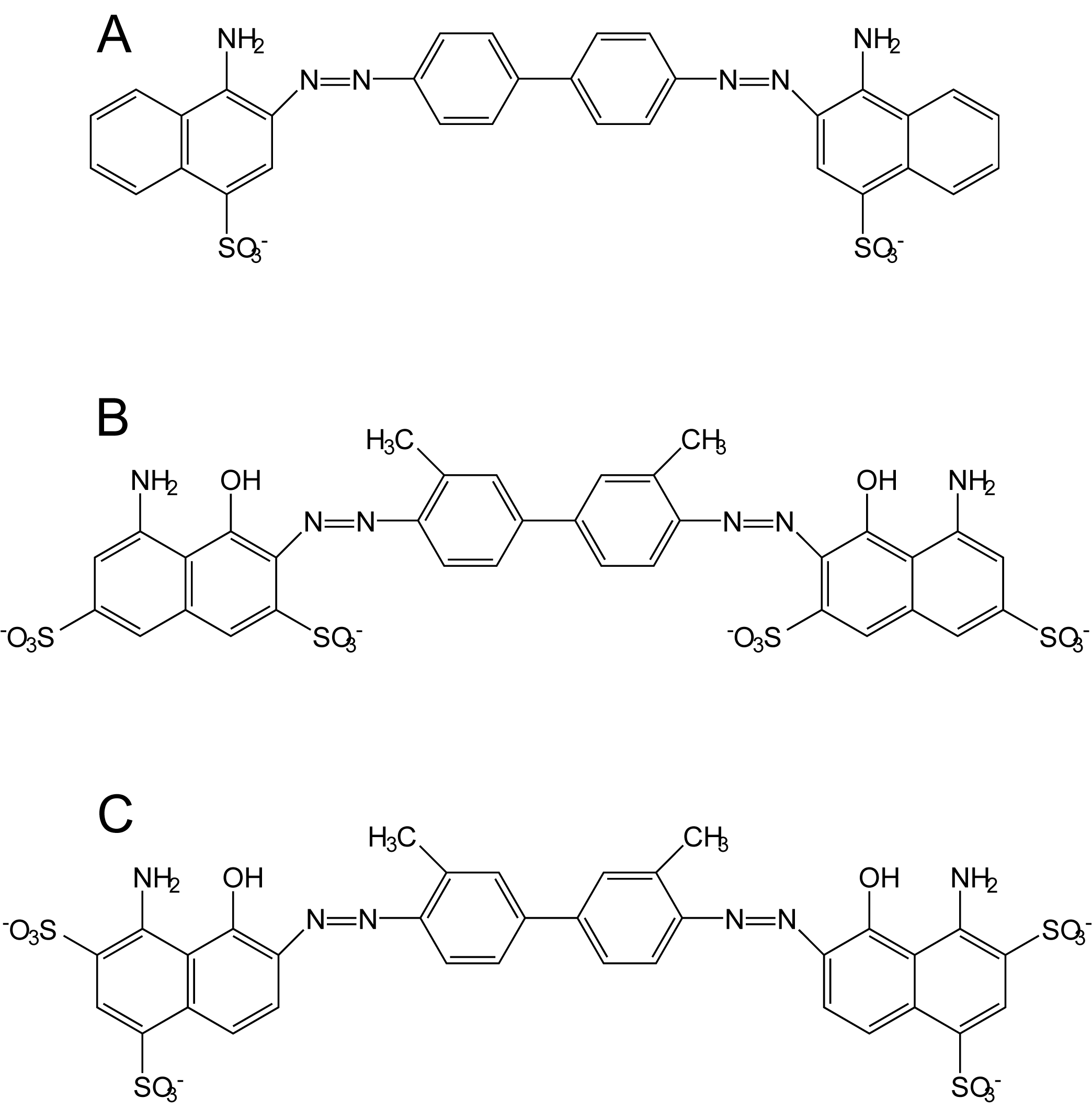

Supplement: Figure S1 — Chemical structures of amyloid imaging fluorophores. (A) Congo red, (B) Trypan blue, and (C) Evans blue. (0.41 MB TIF) [file pone.0002175.s001.tif]

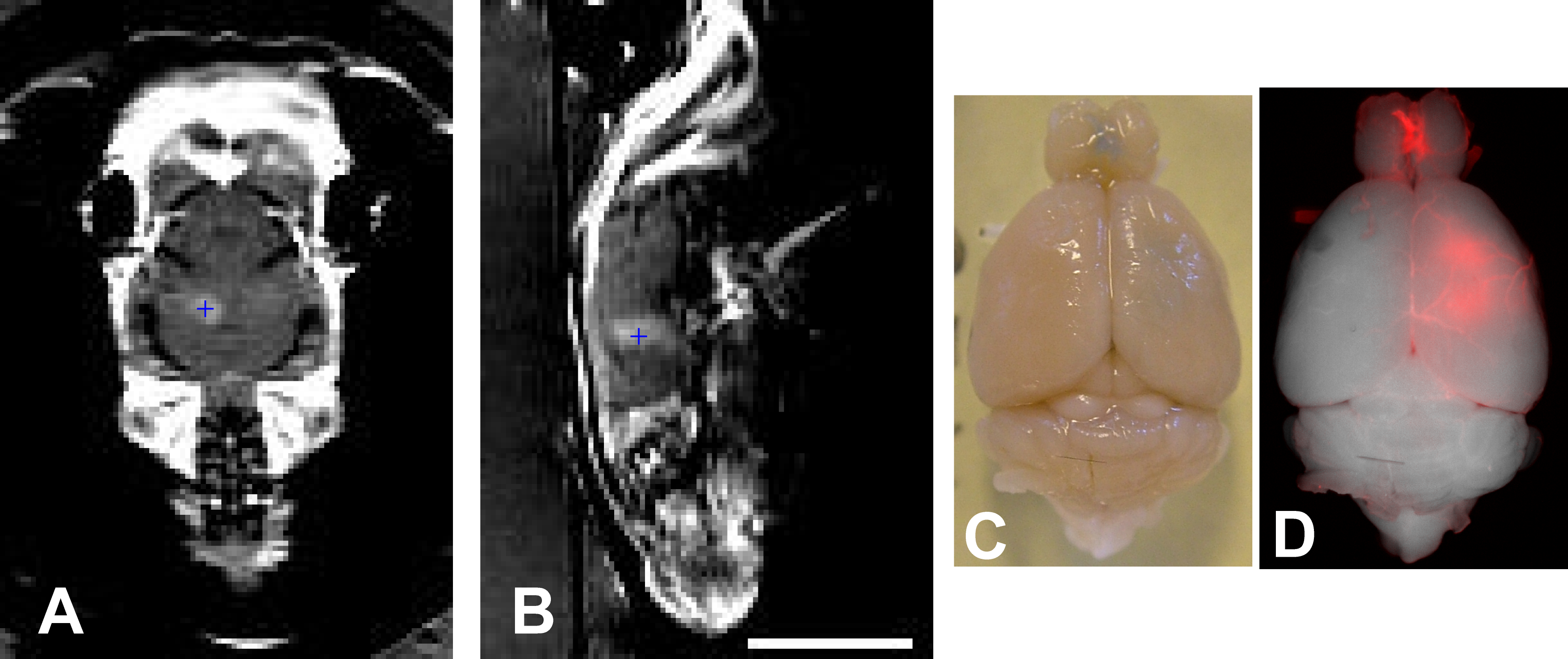

Supplement: Figure S2 — MRI-guided focused ultrasound-microbubble treatment in PDAPP mice. Aged PDAPP mice were treated with FUS-MB at a location determined from pre-treatment MR imaging and received intravenous MR-contrast agent and Trypan blue, an Aβ-targeting red fluorophore, after sonication. (A–B) T1-weighted, contrast-enhanced MR images taken 5 minutes following FUS treatment. Intended sonication locations are indicated by blue “+”. (C–D) Post-mortem brain; the sonication location is faintly visible as a blue spot in right hemisphere on photography (C) and red fluorescence (D) from Trypan blue staining. Scale bar: B, 1 cm. (5.40 MB TIF) [file pone.0002175.s002.tif]

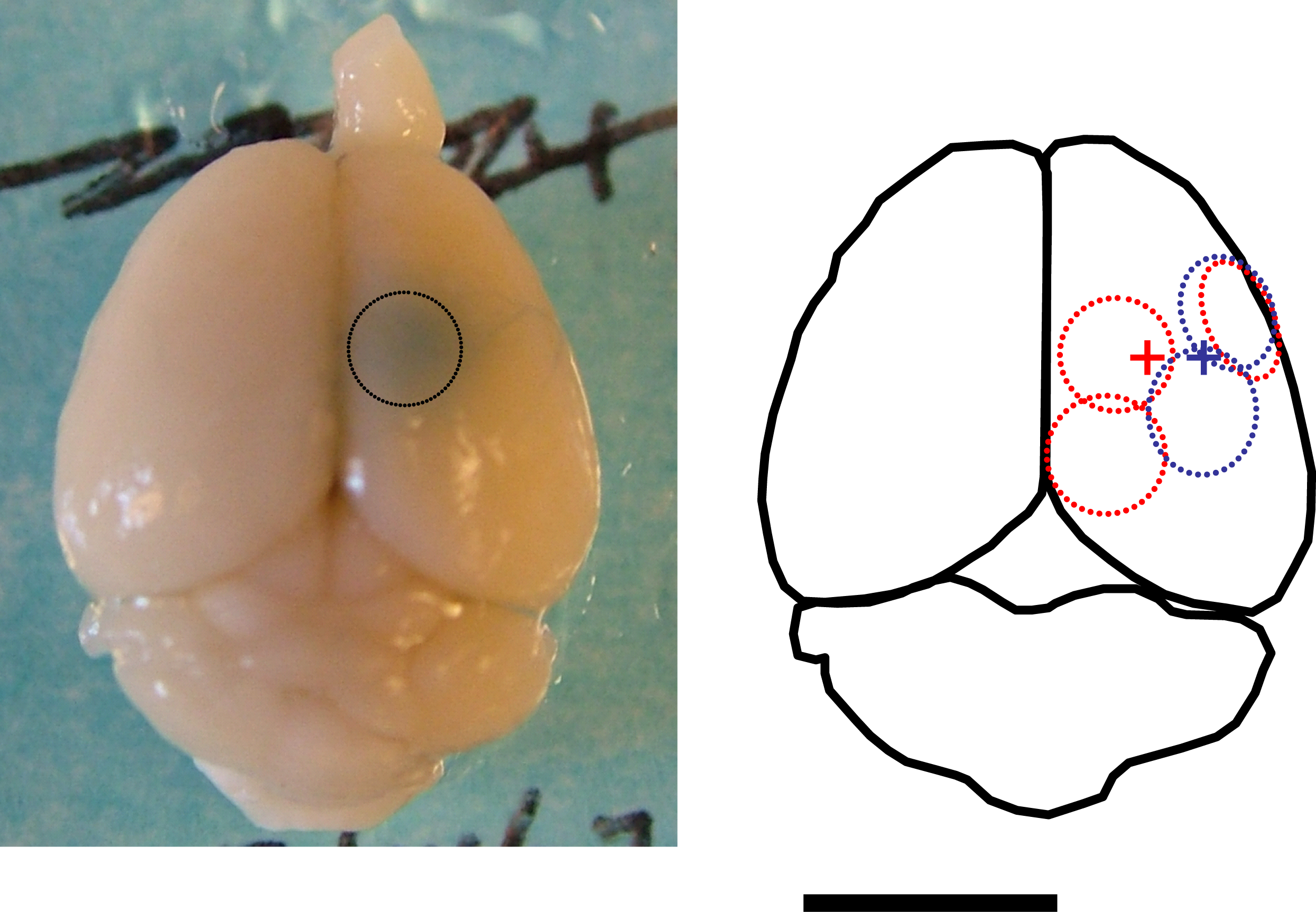

Supplement: Figure S3 — Benchtop focused ultrasound-microbubble treatment. Transgenic mice were sonicated at a single location in the right hemisphere using a benchtop sonication system and received Trypan blue or Evans blue IV. 5/5 animals exhibited focal blue staining in the right hemisphere. (a) Focal blue staining from Trypan blue on post-mortem, excised brain. (b) Schematic displaying sonication locations (red and blue dotted lines) from two different system users (red vs. blue) for two intended target locations (red and blue crosses). Scale bar: B, 1 cm. (5.14 MB TIF) [file pone.0002175.s003.tif]
